# Variation in the *UCP2 *and *UCP3 *genes associates with abdominal obesity and serum lipids: The Finnish Diabetes Prevention Study

**DOI:** 10.1186/1471-2350-10-94

**Published:** 2009-09-21

**Authors:** Titta Salopuro, Leena Pulkkinen, Jaana Lindström, Marjukka Kolehmainen, Anna-Maija Tolppanen, Johan G Eriksson, Timo T Valle, Sirkka Aunola, Pirjo Ilanne-Parikka, Sirkka Keinänen-Kiukaanniemi, Jaakko Tuomilehto, Markku Laakso, Matti Uusitupa

**Affiliations:** 1 University of Kuopio, Department of Clinical Nutrition and Food and Health Research Center, Kuopio, Finland; 2 National Public Health Institute, Department of Health Promotion and Chronic Disease Prevention, Diabetes Unit, Helsinki, Finland; 3 University of Helsinki, Department of General Practice and Primary Health Care, Helsinki, Finland; 4 Vasa Central Hospital, Vasa, Finland; 5 National Public Health Institute, Department of Health and Functional Capacity, Laboratory for Population Research, Turku, Finland; 6 Diabetes Center, Finnish Diabetes Association, Tampere, Finland; 7 Tampere University Hospital, Science Center, Pirkanmaa Hospital District, Tampere, Finland; 8 University of Oulu, Department of General Practice, Oulu, Finland; 9 University of Helsinki, Department of Public Health, Helsinki, Finland; 10 South Ostrobothnia Central Hospital, Seinäjoki, Finland; 11 University of Kuopio, Department of Medicine, Kuopio, Finland; 12 Kuopio University Hospital, Department of Medicine, Kuopio, Finland

## Abstract

**Background:**

We explored the associations of three variants in the uncoupling protein 2 *(UCP2) *gene, one variant in the *UCP2-UCP3 *intergenic region and five variants in the uncoupling protein 3 *(UCP3) *gene with obesity and diabetes related traits in subjects with impaired glucose tolerance participating in Finnish Diabetes Prevention Study. Altogether 507 overweight individuals (body mass index: 31.2 ± 4.5 kg/m^2^, age: 55 ± 7 years) for whom DNA was available were randomized to either an intensified diet and physical activity group or to a conventional care control group.

**Methods:**

We analysed the data from the baseline and annual follow-up visits from years 1, 2 and 3. Measurements of anthropometry, plasma glucose and serum insulin in oral glucose tolerance test, serum total cholesterol, HDL-cholesterol and triglycerides were included. The median follow-up time for type 2 diabetes incidence was 7 years. Genetic variants were screened by restriction fragment length polymorphism or Illumina method.

**Results:**

*UCP3 *gene variant *rs3781907 *was associated with increased serum total and LDL-cholesterol levels, at baseline and during the follow-up period. The same variant was associated with a higher risk of type 2 diabetes. Variants *rs1726745, rs11235972 *and *rs1800849 *in the *UCP3 *gene associated with serum total and LDL-cholesterol at baseline. Haploblock including variants *rs659366, rs653529, rs15763*, and *rs1726745 *was associated with measures of abdominal obesity at baseline and in the longitudinal analysis. The haplotype comprising alleles *rs659366-G, rs653529-A, rs15763-G *and *rs1726745-A *was associated with higher waist-to-hip ratio, and haplotype comprising alleles *rs3781907-G, rs11235972-A*, and *rs1800849-T *was associated with increased serum total and LDL-cholesterol concentrations.

**Conclusion:**

Genetic variation in the *UCP2-UCP3 *gene cluster may act as a modifier increasing serum lipid levels and indices of abdominal obesity, and may thereby also contribute to the metabolic aberrations observed in obesity and type 2 diabetes.

## Background

UCP2 is a member of the mitochondrial inner membrane carrier family that is expressed in a wide variety of tissues, for example in adipose tissue, skeletal muscle and pancreatic islets. The function of UCP2 is tissue-dependent, and its potential roles include the regulation of fat metabolism directly and indirectly, e.g. via effects on insulin secretion [1]. It also has a role in the limitation of reactive oxygen species (ROS) and macrophage-mediated immunity [2]. Recent studies have established UCP2 as a key component of beta cell glucose sensing, since it seems to regulate glucose-stimulated insulin secretion [1,3], and is also a critical link between obesity, beta cell dysfunction and type 2 diabetes (T2DM) [4]. *UCP2 *and *UCP3 *genes are located on chromosome 11q13 adjacent to one another [5,6]. In a recent meta-analysis of genome-wide linkage studies, suggestive evidence for chromosome 11q13.3-22.3 was observed for body mass index (BMI) -defined obesity [7].

There are three common variants in the *UCP2 *gene, one located in the promoter region (*-866 GA, rs659366*), one is a missense variant in exon 4 (*Ala55Val, CT*, *rs660339*), and one locates in the untranslated exon 8 (45 bp *DelIns *in the 3'UTR). Their association with traits related to obesity, energy expenditure or T2DM remains controversial due to inconsistent findings [8-19]. Studies on *rs659366 *have demonstrated associations between the *A *allele and enhanced adipose tissue mRNA expression and decreased risk of obesity [17], increased energy expenditure [20], reduced beta cell function and higher risk of T2DM [14], lower insulin secretion [15], decreased lipid oxidation [16], increased preclinical atherosclerosis in women [21], and increased coronary heart disease risk [18]. Krempler et al. [14] have shown that the functional *rs659366 *variant, located in a multifunctional *cis*-regulatory site, acts as a binding site for a pancreatic transcription factor PAX6. Wang et al. [13] found that the heterozygous combination of the three variants (*-866 GA, Val55Val, DelIns*) was associated with increased BMI, triglyceride and fasting insulin levels.

While the *UCP2 *gene is expressed in almost all tissues, expression of the *UCP3 *gene is mostly restricted to skeletal muscle and brown adipose tissue. Suggested functions of UCP3 include regulation of fatty acid metabolism, redox state, and ROS formation [22,23]. UCP3 seems to be involved in the protection of mitochondria against lipotoxicity [24]. The human *UCP3 *gene gives rise to two main alternative transcripts, the shorter one having a polyadenylation site in intron 6, which terminates approximately 50% of the transcripts. Therefore, human *UCP3 *exists as long (UCP3L) and short (UCP3S) forms [19]. A promoter region variant *-55 CT *(*rs1800849*) is potentially interesting since it is located only 6 bp apart from the TATA box and 4 bp from a DR1 site, which is a part of a retinoic acid response element [25]. In Pima Indians the *rs1800849-T *allele increased UCP3 mRNA expression in skeletal muscle compared with the *C *allele [25], and the expression level correlated negatively with BMI [26]. A direct correlation between sleeping metabolic rate and 24 h energy expenditure and the expression of UCP3 mRNA was also found in Pima Indians [26]. Moreover, the *rs1800849-T *allele was associated with higher waist-to-hip ratio (WHR) [27], decreased risk of T2DM, and atherogenic lipid profile [28], but associations with BMI were controversial [29-32]. No association between *rs1800849 *and body weight was seen in several studies [25,33-36].

The aim of this study was to assess the impact of five previously unexplored variants in addition to the four above-mentioned known genetic variants in the *UCP2-UCP3 *gene cluster, either individually or as haplotypes, on obesity and T2DM related traits. The study subjects with impaired glucose tolerance (IGT) were prospectively followed in the Finnish Diabetes Prevention Study (DPS) [37,38].

## Methods

### Subjects and research design

The DPS is a randomised, controlled, multicenter study carried out in Finland in 1993-2000. The DPS study design and methods used have been reported in detail elsewhere [37-39]. The main inclusion criteria were BMI over 25 kg/m^2^, age 40 to 64 years, IGT based on the mean values of two oral glucose tolerance tests (OGTTs). A total of 522 individuals with IGT were randomised into either a control group or an intensive, individualised diet and physical exercise intervention group stratified according to the clinic, sex, and the mean plasma glucose concentration two hours after an oral glucose load (7.8 to 9.4 or 9.5 to 11.0 mmol/l). DNA was available from 507 individuals (166 men and 341 women). Their mean BMI was 31.2 ± 4.5 kg/m^2 ^and age 55.3 ± 7.1 years. At the baseline, 5% of the individuals were using cholesterol lowering medication. The study protocol was approved by the Ethics Committee of the National Public Health Institute in Helsinki, Finland, and the study participants gave written informed consent. We certify that all applicable institutional and governmental regulations concerning the ethical use of human volunteers were followed during this research.

### Measurements

A medical history was taken and a physical examination done at baseline and at each annual follow-up visit [38]. In this study, measurements from baseline to the 3-year examination were used, including height, weight, waist circumference (WC), hip circumference, serum lipid levels, and 2 h OGTT with glucose and insulin levels before (0 min) and after a 75 g glucose load (120 min) [38]. Plasma glucose was measured at each centre by standard methods. The serum insulin concentration was measured in a central laboratory by a radioimmunoassay method (Pharmacia, Uppsala, Sweden). The intra-assay coefficient of variation was 5.3% and the interassay coefficient of variation was 7.6%. Homeostasis model for insulin resistance (HOMA-IR) was calculated using the formula: fasting plasma glucose (mmol/l) × fasting serum insulin (mU/l)/22.5, and homeostasis model for insulin secretion (HOMA-IS) was calculated as 20 × fasting serum insulin (mU/l)/(fasting plasma glucose [mmol/l] - 3.5) [40]. Serum levels of total cholesterol, high-density lipoprotein (HDL) cholesterol and triglycerides were measured by enzymatic assay in the central laboratory in Helsinki. Formula of Friedewald [41] was used to calculate the concentration of low density lipoprotein (LDL) cholesterol.

### DNA analysis

The single nucleotide polymorphisms (SNP) for genotype analysis were selected from the region spanning the *UCP2 *and *UCP3 *genes (~34.4 kb) by using the International HapMap database and Tagger software [42]. *Rs660339, rs659366*, and *rs1800849 *were forced in the selection procedure. The SNPs covered 86.2% of the genetic information of the studied region (r2>0.8). It should be noted that the *DelIns *variant of the *UCP2 *gene is not included in the database, since it is not a SNP but a 45 bp insertion.

The *rs659366 *and *rs660339 *variants of the *UCP2 *gene and the *rs1800849 *variant of the *UCP3 *gene were screened by the restriction fragment length polymorphism after digestion with *MluI, HincII *and *HaeIII*, respectively, with minor modifications to previously described methods [9,15,43]. The *DelIns *variant of the *UCP2 *gene was analysed by gel electrophoresis of the PCR-product. The intergenic region variant *rs653529 *and four variants locating in the *UCP3 *gene (*rs15763, rs1726745, rs3781907, rs11235972*) were genotyped by using the custom Golden Gate genotyping reagents and consumables (Illumina Inc, San Diego, CA). Only 501 (*rs1726745*) or 502 (*rs653529, rs15763, rs3781907, rs11235972*) subjects were successfully genotyped by Illumina. For other variants, n = 507.

### Statistical analysis

The data were analysed using the SPSS/WIN program version 14.0 (SPSS, Chigago, IL, USA). The normality of distributions of study variables was evaluated with the Kolmogorov-Smirnov test with Lilliefors' correction, and appropriate transformation was used when necessary. For variables with skewed distribution, Kruskal-Wallis test was used. Univariate analysis of variance was used to compare the effect of the gene variants on continuous variables. Adjustment for age, gender and BMI was done, when appropriate. In addition, serum lipoprotein and lipid concentrations were adjusted for the use of cholesterol-lowering medication as well. Chi square test was used in comparison of categorical variables. The relative changes in HOMA-IS from baseline to three years were calculated as follows: [(parameter _3-year _- parameter_baseline_)/parameter_baseline_] × 100%. Longitudinal changes were examined using repeated measures of General Linear Model. Homogeneity of variances was tested using Levene's test. Cox regression analysis, adjusted for the study group, baseline weight, weight change and baseline fasting plasma glucose, was performed to evaluate whether the gene variants predicted the development of T2DM.

Linkage disequilibrium (LD) statistics were calculated by Haploview software [44] and haplotype analysis was done by THESIAS 3.1 [45], which is based on the stochastic-EM algorithm. Haplotype analyses of the quantitative variables were adjusted for age, gender and BMI, when appropriate. The survival analysis for haplotypes was adjusted for the study group, baseline weight, weight change and baseline fasting plasma glucose.

A *p-*value < 0.05 was considered statistically significant. Correction for multiple hypothesis testing was performed with false discovery rate (FDR) using Q-value 1.0 software. π_0 _was estimated with bootstrap method [46] using λ range from 0 to 0.9 by 0.05. Due to the distribution of *p*-values, the λ was set to 0 for correcting the results of Cox regression. Essentially, this is a conservative way of calculating FDR and thereby produces the estimate implicit in the Benjamini and Hochberg methodology. In text, *q *stands for FDR, and is reported for each *p *< 0.05 and should be interpreted as minimum FDR that is incurred when calling that test significant. Data are given as means ± SD, unless otherwise indicated.

## Results

### Genotype and allele frequencies and LD

**Figure 1:**
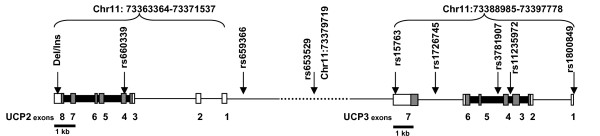
**Schematic representation of the *UCP2 *and *UCP3 *genes indicating the locations of the analysed variants**. Grey boxes, coding region; white boxes, UTR; dotted line, intergenic region of ~17 kb.

The genotype frequencies of the nine gene variants were consistent with the Hardy-Weinberg equilibrium (*p*-values ranging from 0.065 to 0.952), and did not differ between the study groups. Location of the variants in the *UCP2 *and *UCP3 *genes and their minor allele frequencies and LD statistics (D' and r^2^) are presented in Figure 1 and Table 1. D' values were between 0.053 and 1.0, whereas r^2 ^varied from 0.002 to 0.979. Although variants *rs11235972 *and *rs1800849 *were in strong linkage disequilibrium (D' = 1.0 and r^2 ^= 0.979), no completely redundant variants were observed; thus all nine variants were included in subsequent association analysis. Based on solid spine of LD, haplotype analysis was done separately for three haplotype blocks, block 1 including *DelIns *and *rs660339 *variants of the *UCP2 gene*, block 2 including *rs659366, rs653529, rs15763 *and *rs1726745 *of the *UCP2 *and *UCP3 *genes, and block 3 covering variants *rs3781907, rs11235972 *and *rs1800849 *of the *UCP3 *gene.

**Table 1:** Pairwise linkage disequilibrium, presented as D' and r^2 ^values, among the nine variants in *UCP2 *and *UCP3 *genes (haploblocks are shown bolded)

	**SNP ID**	**D'**								
		
		***DelIns***	***rs660339***	***rs659366***	***rs653529***	***rs15763***	***rs1726745***	***rs3781907***	***rs11235972***	***rs1800849***
r^2^	*DelIns* (0.297)		**0.977**	0.684	0.630	0.906	0.402	0.829	0.856	0.857
	*rs660339 CT *(0.470)	**0.451**		0.985	0.912	0.862	0.548	0.282	0.056	0.053
	*rs659366 GA *(0.403)	0.292	0.736		**0.952**	**0.867**	**0.844**	0.218	0.133	0.134
	*rs653529 AG *(0.415)	0.236	0.663	**0.863**		**0.902**	**0.805**	0.243	0.119	0.125
	*rs15763 GA *(0.218)	0.542	0.232	**0.309**	**0.318**		**1.0**	0.871	0.934	0.933
	*rs1726745 GA *(0.403)	0.046	0.181	**0.325**	**0.311**	**0.188**		0.756	0.948	0.940
	*rs3781907 AG *(0.327)	0.140	0.034	0.016	0.020	0.102	0.189		**0.804**	**0.807**
	*rs11235972 GA *(0.371)	0.181	0.002	0.015	0.012	0.142	0.358	**0.535**		**1.0**
	*rs1800849 CT *(0.377)	0.185	0.002	0.016	0.013	0.145	0.360	**0.527**	**0.979**	

Major/minor alleles, and the frequencies of the minor alleles of the corresponding variants are indicated next to each variant identification number

### Baseline characteristics of subjects

No significant differences were observed among the genotypes regarding body weight, BMI, fasting and 2-h insulin levels, HOMA-IR or HOMA-IS. Fasting and 2-h glucose levels differed among the *rs660339 *genotypes (*p/q *0.006/0.006 and 0.025/0.010, respectively), and among the *rs659366 *genotypes (*p/q *0.007/0.006 and 0.025/0.010, respectively). Specifically, the *rs660339-CC *and *rs659366-AA *homozygotes had the highest fasting glucose concentrations and the *rs660339-CC *and *rs659366-GG *homozygotes had the highest 2-h glucose concentrations. In addition, *rs653529 *associated with 2-h glucose level, so that the individuals with *AA*-genotype had the highest values (*p/q *0.028/0.010), but the associations with glucose were not dependent on the allele dosage.

Four gene variants in the haploblock 2 associated with indices of abdominal obesity, namely WC, WHR and waist-to-height ratio (WHtR). *Rs659366, rs653529, rs15763 *and *rs1726745 *associated with WHR at baseline (*p/q *0.048/0.014, 0.009/0.006, 0.018/0.010 and 0.031/0.010, respectively), as subjects with *rs659366-AA, rs653529*-*GG*, *rs15763*-*AA *and *rs1726745*-*GG *genotypes had the lowest WHR (Table 2). Although less consistent, associations were also seen between these variants and WC and WHtR. WC was associated with *rs659366, rs653529 *and *rs1726745*, whereas WHtR was associated with *rs653529, rs15763 *and *rs1726745 *(Table 2). Also the *DelIns *variation in *UCP2 *associated with WHR, subjects with the *DelDel *genotype having the highest WHR at baseline (*p/q *0.017/0.256).

**Table 2:** Associations of representative SNPs in the *UCP2-UCP3 *gene region with indices of abdominal obesity at the baseline

		**Genotype**				
				
**SNP**	**Obesity index**	**Homozygous for the common allele**	**Heterozygous**	**Homozygous for the rare allele**	***p*^a^**	***q***
*rs659366*	WC, cm	102 ± 11	101 ± 11	100 ± 11	0.033	0.010
	WHR	0.93 ± 0.07	0.92 ± 0.07	0.91 ± 0.08	0.048	0.014
	WHtR	0.61 ± 0.06	0.61 ± 0.06	0.61 ± 0.07	0.119	0.027

*rs653529*	WC, cm	102 ± 11	101 ± 11	100 ± 11	0.011	0.007
	WHR	0.93 ± 0.07	0.92 ± 0.07	0.91 ± 0.08	0.009	0.006
	WHtR	0.61 ± 0.06	0.61 ± 0.06	0.60 ± 0.07	0.022	0.010

*rs15763*	WC, cm	102 ± 11	100 ± 10	100 ± 12	0.075	0.019
	WHR	0.92 ± 0.07	0.92 ± 0.07	0.90 ± 0.07	0.018	0.010
	WHtR	0.61 ± 0.07	0.60 ± 0.06	0.60 ± 0.07	0.024	0.010

*rs1726745*	WC, cm	100 ± 11	102 ± 11	102 ± 11	0.008	0.006
	WHR	0.91 ± 0.08	0.92 ± 0.07	0.93 ± 0.07	0.031	0.010
	WHtR	0.60 ± 0.06	0.62 ± 0.06	0.61 ± 0.07	0.003	0.006

Data are means ± SD

^a^Univariate ANOVA, adjusted for age, gender, BMI

WC, waist circumference; WHR, waist-to-hip ratio; WHtR, waist-to-height ratio

*Rs1726745, rs3781907, rs11235972 *and *rs1800849 *were associated with serum total cholesterol and LDL-cholesterol levels. Subjects with *rs1726745*-*GG*, *rs3781907*-*GG*, *rs11235972*-*AA *and *rs1800849-TT *genotypes had the highest serum total cholesterol concentrations among the variants (*p/q *0.022/0.010, 0.005/0.006, 0.032/0.010 and 0.050/0.014, respectively), as well as serum LDL-cholesterol (*p/q *0.005/0.006, 0.004/0.006, 0.045/0.014 and 0.071/0.018, respectively). Moreover, the total cholesterol-to-HDL-cholesterol ratio was highest for the subjects with *rs3781907*-*GG *genotype (*p/q *0.045/0.013). Baseline characteristics of the DPS subjects according to *rs3781907 *are presented in the Table 3. Regarding *rs3781907 *and *rs11235972*, the results on lipids and lipoproteins remained unchanged, if the individuals using cholesterol lowering medication (n = 25) were excluded from the analyses. Regarding *rs1726745*, and *rs1800849*, the results were statistically significant only for *rs1726745 *association with LDL-cholesterol, if the individuals using cholesterol lowering medication were excluded.

**Table 3:** Baseline characteristics and conversion to type 2 diabetes (T2DM) according to the genotypes of SNP *rs3781907 *at *UCP3 *gene

	**Genotype**				
			
	***AA***	***AG***	***GG***	***p*^b^**	***q***
n (M/F)^a^	221 (73/148)	233 (79/154)	48 (13/35)	0.645	0.714
Age (years)	56 ± 7	55 ± 7	53 ± 7	0.077^c^	0.421
Weight (kg)	86.3 ± 14.6	85.8 ± 13.5	87.3 ± 15.9	0.878	0.924
BMI (kg/m^2^)	31.3 ± 4.8	31.1 ± 4.2	31.6 ± 5.2	0.991	0.954
Waist-to-hip ratio	0.92 ± 0.07	0.92 ± 0.07	0.93 ± 0.08	0.583	0.067
Fasting plasma glucose (mmol/l)	6.2 ± 0.8	6.1 ± 0.7	6.2 ± 0.7	0.295	0.047
Fasting serum insulin (pmol/l)	85 ± 40	92 ± 51	86 ± 30	0.665^c^	0.070
Serum total cholesterol (mmol/l)	5.5 ± 0.9	5.7 ± 0.9	5.9 ± 1.0	0.005	0.006
Serum LDL-cholesterol (mmol/l)	3.5 ± 0.8	3.7 ± 0.8	3.9 ± 1.0	0.004	0.006
Serum HDL-cholesterol (mmol/l)	1.22 ± 0.29	1.22 ± 0.30	1.17 ± 0.25	0.834	0.081
Serum triglycerides (mmol/l)	1.69 ± 0.73	1.74 ± 0.83	1.81 ± 0.70	0.558	0.066
Total cholesterol-to-HDL-cholesterol ratio	4.73 ± 1.22	4.90 ± 1.26	5.15 ± 1.15	0.045	0.013
Converters to T2DM (n/%)^a^	72/33%	89/38%	23/48%	0.039	0.394

Data are means ± SD

^a^χ^2 ^test

^b ^Univariate ANOVA, adjusted for age and sex (anthropometric measurements), for age, sex and BMI (glucose, insulin), or for age, sex, BMI, and the use of cholesterol lowering medication (lipids)

^c ^Kruskal-Wallis test

### The 3-year changes

**Figure 2:**
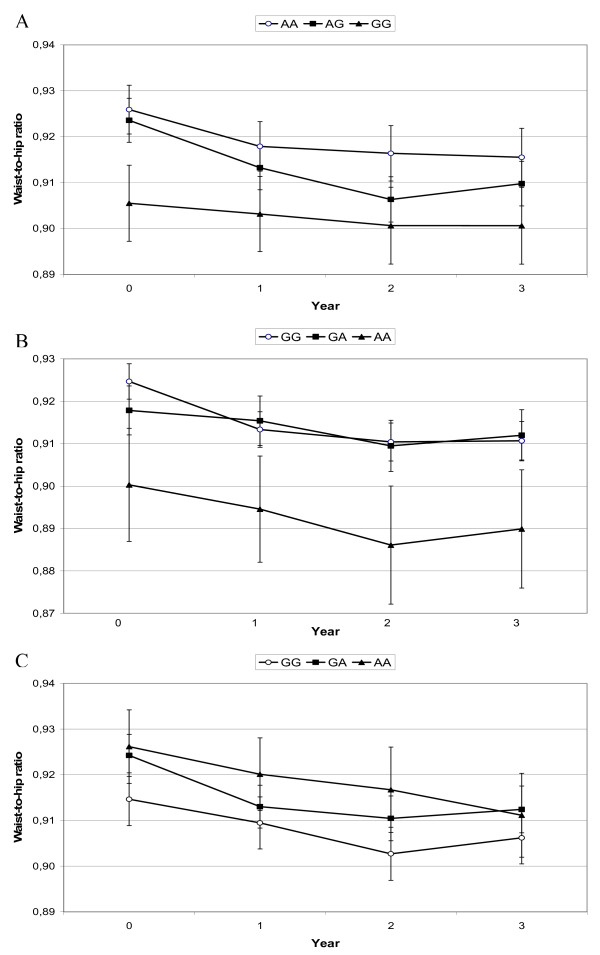
**Waist-to-hip ratio from baseline to year 3 according to *rs653529 *(a), *rs15763 *(b), and *rs1726745 *(c) variants, *p/q *0.025/0.030, 0.039/0.030 and 0.035/0.030, respectively**. Data are mean ± SEM.

Longitudinal analysis of WHR showed that subjects with *rs653529-GG*, *rs15763*-*AA *and *rs1726745*-*GG *genotypes had the lowest WHR throughout the 3-year follow-up (*p/q *0.025/0.030, 0.039/0.030 and 0.035/0.030, respectively) (Figure 2). Longitudinal analyses of WC and WHtR showed similar results, with WC being associated with *rs15763 *(*p/q *0.030/0.030) and WHtR associating with *rs15763 *and *rs1726745 *(*p/q *0.009/0.030, 0.040/0.030, respectively).

**Figure 3:**
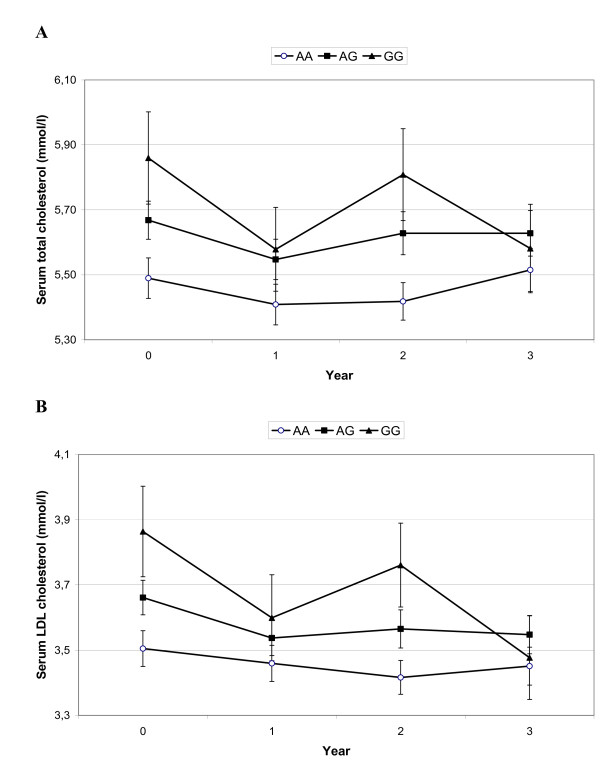
**Serum total cholesterol (a) and LDL-cholesterol (b) levels, from baseline to year 3, according to *rs3781907 *variant of the *UCP3 *gene, *p/q *0.020/0.030 and 0.010/0.030, respectively**. Data are mean ± SEM.

Longitudinal changes in serum cholesterol levels were associated with *rs3781907*-*AA *genotype having the lowest total cholesterol (*p/q *0.020/0.030) and LDL-cholesterol (*p/q *0.010/0.030) throughout the years 0-3 (Figure 3). Subjects with *rs3781907-AA *genotypes also had the lowest serum triglyceride levels, but this was seen only in the control group (*p/q *0.039/0.030). HDL-cholesterol level was lowest among the subjects with *rs3781907-GG *genotype in the intervention group (*p/q *0.041/0.030). Furthermore, the total cholesterol-to-HDL cholesterol ratio was highest among subjects with the *rs3781907-GG *genotype in the entire DPS (*p/q *0.015/0.030). All these results remained significant if the individuals using cholesterol lowering medication were excluded.

The 3-year change in HOMA-IS was associated similarly with all three *UCP2 *variants (*DelIns, rs660339, rs659366*), the intergenic region variant *rs653529 *and *UCP3 *variant *rs15763*, showing increased values for wild-type subjects, intermediate for heterozygous and the lowest values for homozygous subjects (*p/q *0.004/0.304, 0.016/0.325, 0.037/0.325, 0.018/0.325 and 0.016/0.325, respectively), whereas no association with other four *UCP3 *variants was found.

### Haplotype analysis

In order to confirm the associations with serum lipids and WHR, haplotype analysis was performed separately for blocks 1, 2, and 3. Only the haplotypes with frequency ≥ 0.05 were included in the analysis; thus, block 1 consisted of 3 haplotypes (*DelC, InsT*, and *DelT*), block 2 of 4 haplotypes (*GAGA, GAGG, AGAG*, and *AGGG*) and block 3 of 3 different haplotypes (*AGC, GAT*, and *AAT*) (Table 4).

**Table 4:** Frequencies and associations of the major haplotypes (frequency ≥ 0.05) of three haploblocks in the *UCP2*-*UCP3 *gene region

**Haploblock**	**Marker**				**Frequency**	**Association**
Block 1	*DelIns*	rs660339				
	*Del*	*C*			0.519^a^	NS
	*Ins*	*T*			0.300	NS
	*Del*	*T*			0.177	NS

Block 2	rs659366	rs653529	rs15763	rs1726745		
	*G*	*A*	*G*	*A*	0.359^a^	NS
	*G*	*A*	*G*	*G*	0.203	NS
	*A*	*G*	*A*	*G*	0.202	↓ WHR, *p *= 0.050^b^
	*A*	*G*	*G*	*G*	0.175	NS

Block 3	rs3781907	rs11235972	rs1800849			
	*A*	*G*	*C*		0.584^a^	NS
	*G*	*A*	*T*		0.288	↑ cholesterol, *p *= 0.006^c^ ↑ LDL, *p *= 0.024^c^
	*A*	*A*	*T*		0.084	↓ΔLDL, *p *= 0.037^c^

^a ^The most frequent haplotype is the reference, with which the others are compared

^b ^Adjusted for age and gender

^c ^Adjusted for age, gender and BMI

↑, higher in comparison with the reference haplotype; ↓, lower in comparison with the reference haplotype

In block 2 the haplotype *AGAG *showed lower WHR at baseline when compared with the reference haplotype *GAGA *(*p *= 0.050). In block 3 the haplotype *GAT *had higher serum total cholesterol (*p *= 0.006) and LDL-cholesterol (*p *= 0.024) at baseline when compared with the reference haplotype *AGC*. Moreover, the haplotype *AAT *showed a greater decrease (0-3 years) in LDL-cholesterol level compared with the reference haplotype *AGC *during the 3-year follow-up (p = 0.037). No associations were seen for the haplotypes in the haploblock 1.

### Conversion to T2DM

During a median follow-up of seven years 185 individuals (75 in the intervention group and 110 in the control group) developed T2DM. The subjects with the *rs3781907*-*G *allele were at a higher risk for T2DM when compared with subjects with *AA *genotype, with hazard ratio (HR) of 1.48 (95%CI 1.09-2.00), *p*/*q *0.011/0.100. We also analysed the genotype × study group interaction for the diabetes conversion, but it was not statistically significant (*p *= 0.447). The percentage of subjects with *AA *genotype converting to T2DM was 33%, whereas it was 38% and 48% for the subjects with *AG *and *GG *genotypes, respectively (*p/q *0.039/0.394) (Table 3). The percentages of converters were similar for both study groups, with *GG *genotype showing the highest and *AA *genotype showing the lowest percentages of converters.

Surprisingly, subjects in the intervention group carrying the *UCP2 InsIns *genotype were at a higher risk compared with subjects with *Del *allele, with HR 2.53 (95%CI 1.11-5.73), *p*/*q *0.027/0.243. Such a risk increment was not seen in the control group, nor in the entire DPS. No genotype × study group interaction was seen here either.

The conversion to T2DM during the follow-up did not differ significantly among the other gene variants. In the haplotype analysis, none of the haplotypes studied were associated with an increased risk of T2DM.

## Discussion

In the present study we evaluated whether the *UCP2 *and *UCP3 *genes act as modifiers for obesity and diabetes related risk factors. This study provides new evidence concerning associations between genetic variations in the *UCP2 *and *UCP3 *genes and serum lipid concentrations as well as indices of abdominal obesity, both being characteristics of the metabolic syndrome. Previous studies have explored effects of these genes on various traits in several populations, with inconsistent results concerning associations with lipid levels and WHR/abdominal obesity (Table 5). The study subjects in the DPS were overweight with IGT, and thus at a high risk to develop T2DM. The advantages of this study population are i) homogenous, carefully selected and phenotyped subjects, ii) prospective study design with extensive longitudinal follow-up data on key variables, iii) confirmation of new T2DM diagnosis based upon two subsequent OGTTs, and iv) Finnish population with only few founding members and thus genetically relatively homogeneous. On the other hand, one drawback for genetic analyses in the DPS is the limited power due to relatively small sample-size, particularly if the analyses are carried out in smaller sub-groups. In this study, stratification to different groups was unnecessary, since the associations with WHR and lipid levels were observed in the entire DPS study population.

**Table 5:** Associations of *UCP2 *and *UCP3 *gene variants with waist-to-hip ratio (WHR) and serum lipid concentrations in previous studies

	**Ref.**	**Subjects** **(number, ethnicity)**	**Variant**	**Risk allele**	**Association**
**WHR**	[43]	710, South Indian 450, Caucasoid	*UCP3: rs1800849*	*T*	Higher WHR

	[27]	162, Caucasoid	*UCP3: rs1800849*	*T*	Higher WHR

	[34]	460, Asian	*UCP3*: haplotype including *rs1800849*	*C *in haplotype *CGTACC*	Higher WHR

**Lipids**	[28]	1175, Caucasoid	*UCP3: rs1800849*	*T*	Higher total and LDL-cholesterol conc.

	[13]	796, several	*UCP2: rs659366* *UCP2: rs660339* *UCP2: Del/Ins*	Heterozygous genotype combination *GTI/ATD*	Higher triglyceride concentration

	[56]	681, Caucasoid	*UCP2: rs659366*	*A*	Higher triglyceride, total and LDL-cholesterol conc.

	[55]	658, Asian	*UCP2: rs659366* *UCP2: rs660339*	*A* *T*	Lower HDL-cholesterol concentration

	[32]	282, Japanese	*UCP3: rs1800849*	*C*	Lower HDL-cholesterol concentration

To our knowledge, this is the first study exploring effects of the gene variants *rs653529, rs15763*, *rs1726745, rs3781907 *and *rs11235972 *on several metabolic traits. Interestingly, subjects with *rs3781907-G *allele experienced a higher risk of T2DM and dyslipidemia compared with subjects homozygous for the common allele. The conversion to T2DM and serum levels of total cholesterol, LDL-cholesterol and total cholesterol-to-HDL-cholesterol ratio were modified by the dosage of *rs3781907-G *allele. The *UCP3 *promoter variant *rs1800849*, which is located in the same haploblock and is in LD with *rs3781907*, has been previously shown to be associated with an increased [47] or decreased [28] risk of T2DM, increased skeletal muscle UCP3 mRNA expression [25], higher total, LDL- [28] and HDL-cholesterol concentrations [32], higher [29] or lower BMI [30-32], higher WHR [27,43] and higher fat mass and lean mass [48]. In this study *rs1800849 *was associated with higher total cholesterol and LDL-cholesterol concentration at baseline, both as alleles and also as a member of the haploblock 3, in line with findings from earlier studies [28]. Interestingly, all the haploblock 3 variants were associated with total and LDL-cholesterol at the baseline, although the most consistent association with various serum lipoprotein and lipid concentrations at baseline and longitudinally was seen for *rs3781907 *that was also a risk factor for conversion to T2DM. FDR was low for these associations, which further provides support for our findings. However, as no previous studies with this variant exist, a confirmation of the present results is needed in other populations.

Although none of the gene variants were associated with weight or BMI, variants in the haploblock 2 associated with several indices of abdominal obesity at baseline (*rs659366, rs653529, rs15763, rs1726745*) and longitudinally (*rs653529, rs15763, rs1726745*). Although waist circumference and BMI are strongly correlated [49], WC, WHR and WHtR are all independent obesity-related predictors of cardiovascular risk independent of BMI [50], and together with serum lipid concentrations they are simple clinical surrogate markers of excess visceral/ectopic fat [49]. In this study the genetic variation in *UCP2-UCP3 *gene cluster seemed to be also associated with both serum lipids and indices of abdominal obesity as well. The single-marker associations were mostly haploblock specific, so that the markers from haploblock 2 were associated with central obesity indices, whereas the markers from the haploblock 3 were associated with serum cholesterol concentrations. The results of haplotype analyses were in line with the results of single marker analyses, but they did not reveal a haplotype that would explain the associations substantially better than the individual markers. We conclude that *rs1726745 *explains most of the associations (both baseline and longitudinal) seen in haploblock 2 with central obesity, and *rs3781907 *explains most of the associations seen in haploblock 3 with total and LDL-cholesterol concentrations. However, it is difficult to suggest whether the causative variant truly is one of the studied markers or simply a SNP that is in complete disequilibrium with them.

As for the functional *UCP2 *promoter variant *-866 GA *(*rs659366*), previous studies have found that it is associated with a reduced prevalence of obesity [17], reduced risk of coronary artery disease [51], reduced [52] or increased [14] risk of T2DM, lower insulin secretion [15,53], reduced insulin sensitivity [54], a decreased [13] or increased [17]*UCP2 *mRNA level in adipose tissue, higher oxidative stress and risk of coronary heart disease [18], decreased HDL-cholesterol [55], and increased serum triglyceride, total cholesterol and LDL-cholesterol levels [56]. In our study, the subjects with the *rs659366-A *allele had lower WC and WHR at baseline, compared with subjects with *G *allele, both individually as well as a member of haploblock 2. Also the insulin secretion, measured by HOMA-IS, seemed to be decreased most in the subjects with *rs659366-A *allele during the 3-year follow-up, although no differences were seen at the baseline. These findings are in line with previous findings [15,17,53]. FDR was low for the associations with abdominal obesity indices thus supporting it, but high for the associations with HOMA-IS, which of course weakens the strength of this finding.

UCP2 has been said to act as adiposity angel and diabetes devil [57], whereas increased expression of UCP3 has been suggested to be associated with weight loss success [58]. The promoter area variant alleles in *UCP2 (rs659366-A*) and in *UCP3 (rs1800849-T*) have been shown to increase gene expression, and thus these alleles might protect against obesity. In our study the *rs659366-A *allele were associated with lower WHR and WC at baseline, in line with findings by Esterbauer et al. [17]. However, no association between *rs1800849 *and obesity was seen in this study. Neither were the promoter area variants associated with risk of T2DM. This could be due to the relatively small study population, and also the genetic 'makeup' varies from one population to another. Furthermore, as the mean BMI of the study population was 31.2 kg/m^2^, the 'obese character' may also have masked the effects of these genetic variants, which may explain the lack of association with obesity.

## Conclusion

In summary, several novel findings were detected and they need to be confirmed. The subjects with the *rs3781907-G *allele had higher levels of serum total and LDL-cholesterol concentrations at baseline and at longitudinal follow-up, and a higher risk of T2DM, when compared with the subjects with *A*-allele. The subjects with *rs1726745*-*G*, *rs11235972*-*A *and *rs1800849-T *alleles had also higher serum total and LDL-cholesterol concentrations compared with those carrying the more common alleles. Several variants (*rs659366-AA, rs653529*-*GG*, *rs15763*-*AA *and *rs1726745*-*GG*) were associated with lower abdominal obesity indices, at baseline and longitudinally.

## Competing interests

The authors declare that they have no competing interests.

## Authors' contributions

TS participated in designing and performing the genotyping and statistical analysis and drafted the manuscript; LP participated in designing the genetic studies and haplotype analysis and writing the manuscript; AMT and MK participated in statistical analysis and revising the manuscript; JL, JGE, TTV, SA, PIP, SKK, and ML contributed to study design and coordination and revised the manuscript; JT and MU are the principal investigators of the study and participated in writing the manuscript. All authors read and approved the final manuscript.

## Pre-publication history

The pre-publication history for this paper can be accessed here:


